# The Risks of Patient Pill Splitting: A Case Report and Review

**DOI:** 10.7759/cureus.48610

**Published:** 2023-11-10

**Authors:** James Espinosa, Gabriel Meister, Mohammad Rattu, Alan Lucerna

**Affiliations:** 1 Department of Emergency Medicine, Jefferson Health, Stratford, USA; 2 School of Pharmacy, Temple University, Philadelphia, USA

**Keywords:** emergency medicine and pill splitting, emergency medicine and tablet splitting, medication safety, tablet splitting, pill splitting

## Abstract

The cutting or splitting of pills can be used by patients to create an intermediate dose or to save money in situations where a higher dose is priced at relatively the same cost as a lower dose. A number of studies of selected medications have concluded that pill splitting in general can be done without adverse effects, with the exception of enteric-coated medications and extended-release medications. Individual patients should be assessed for evidence of patient understanding, as well as the physical abilities for pill splitting. Here, we present the case of a patient whose lack of understanding and inability to organize the pill-splitting process led to poor control of her hypertension, resulting in an emergency department (ED) evaluation.

## Introduction

There are a number of caveats concerning pill splitting, one of which is an appropriate level of patient understanding of the process [1,2]. Here, we present the case of a patient in which pill splitting resulted in an elevation of blood pressure that was not immediately life-threatening but was very anxiety-producing to the patient. This case report was presented in poster form at the Rowan Research Day, which was held on May 4, 2023, in Stratford, New Jersey.

## Case presentation

An 86-year-old female who lived alone presented to the emergency department (ED) with a complaint that her blood pressure when taken on a home blood pressure monitor had been higher than her baseline for approximately one month. She denied any somatic complaints. She had a past medical history that was significant for hypertension. Her only medication was stated to be lisinopril 20 mg per day. Her blood pressure on arrival to the ED was 180/100 mm Hg. Her vital signs were otherwise within normal limits. The patient's physical examination was unremarkable. Basic laboratory studies including a complete blood count, basic metabolic panel, troponin, and urinalysis were all within normal limits. An electrocardiogram was within normal limits. The patient showed the treatment team a written log of her home blood pressure readings and pointed out that the readings were essentially very close to 130/80 mm Hg until approximately 30 days prior to the ED visit.

On inspection of her actual medications, multiple bottles of lisinopril were noted. There was a bottle of lisinopril pills marked 40 mg strength. There was also a bottle of 40 mg pills that had been clearly split in half to 20 mg strength, as well as a bottle with smaller-sized pills that had been split. These pills were, on close inspection, 10 mg (splits) of the 20 mg doses.

On further questioning, it became clear that the patient had been taking 20 mg of lisinopril for over a year with stable blood pressure readings. She had heard from a friend that it would be less expensive if she requested 40 mg pills and split them into 20 mg pills. About a month prior to the ED visit, she requested that her primary physician reorder her lisinopril as 40 mg, with the understanding that she would split them into 20 mg pills. She reported that she started splitting the pills at that time. She used a pill-splitting tool that she had purchased at a pharmacy. In reference to the 10 mg pills, she stated that she believed that she mixed up the 20 mg split pills and the 40 mg pills, producing the resulting 10 mg pills. She recalls that she had been, in fact, mostly taking the smaller (10 mg) pills for the last month. The patient was given lisinopril 10 mg by mouth while in the ED. Her blood pressure improved. Her primary care provider was contacted by the ED team. The patient agreed to discard the 10 mg and 40 mg pills and to return to taking 20 mg of lisinopril once a day. A prescription was written for the 20 mg strength. The patient was discharged with a ride home. The primary care provider agreed to have the patient see a social worker who was associated with the office to review her insurance status and to assess what economic impact, if any, the pill splitting would have on the patient's particular prescription.

The patient followed up with the primary care physician in two days where her blood pressure was 130/82. On telephone contact three weeks later, the patient noted that her blood pressure had remained in the 130/80 range on her home blood pressure monitor and that she would follow up with her primary care provider in one month. She had stopped pill splitting at the time of the ED visit.

## Discussion

The cutting or splitting of pills can be used by patients to create an intermediate dose or to save money in situations where a higher dose is priced at relatively the same cost as a lower dose.

Some patients state that they are splitting pills in order to save money. An analysis of data from a large dataset derived from a large managed care plan concluded that for certain selected medications, pill splitting can be a cost-reduction practice [1]. This savings potential extends to several psychotropic medications [2].

A number of studies of selected medications have concluded that pill splitting can be done without adverse effects. Freeman et al. [3] and Freeman et al. [4] concluded that the appropriateness of pill splitting should be determined for individual medications by individual patients. Individual patients should be assessed for evidence of patient understanding and physical abilities for pill splitting. Noviasky et al. found no decrease in the effect of comparable split tablets of a number of statin medications [5].

A systematic review concluded that there is little overall evidence to support safety concerns concerning pill splitting, with the exception of extended-release tablets and anyone with physical or intellectual limits who may have difficulty with the mechanics of pill splitting [6].

Pill splitting should be done safely and accurately. A number of researchers have concluded that the use of a commercially available pill-spitting device is more accurate than hand splitting with a knife [7]. Scored pills are easier to split than non-scored pills. The safety of splitting should be discussed with the primary care physician and the patient's pharmacist [8].

Not all pills can or should be split, based on the characteristics of the pill and the patient. A consensus of common sense ideas from the literature appears to be that if a pill crumbles, it is not a good target for splitting. Non-scored pills are not good candidates for pill splitting. The patient should be able to split the pill, taking into account visual acuity, hand-eye coordination, and strength. Pill splitting should not make the regimen excessively complicated [9].

If pill splitting is advised by the primary care provider, the patient should be cautioned not to use a knife or razor blade. A commercially available pill splitter should be used [7,9].

## Conclusions

Pill splitting can be used by patients to create an intermediate dose or to save money where a higher dose is priced at relatively the same cost as a lower dose. Safe pill splitting by a patient requires, first and foremost, the appropriate level of visual acuity and strength to split the pill, as well as the ability to cognitively understand the proper storage and use of the split pills. If a pill crumbles, it is not a good target for splitting. Tablets and pills that are film-coated to reduce an unpleasant taste are not good candidates for splitting. If the pill is not scored, pill splitting is more difficult. Extended-release and enteric-coated pills, as well as critical dose products with narrow therapeutic windows, are not good targets for pill splitting. Pill splitting should not make the regimen excessively complicated. These issues speak to the need for patients to discuss pill-splitting questions with their primary care physician and their pharmacist.
